# Role of TRPV4 in the Diagnosis and Treatment of *Helicobacter pylori* Infection in Children with Duodenal Ulcers

**DOI:** 10.1155/2022/2777882

**Published:** 2022-01-04

**Authors:** Chuanying Li, Rong Cheng, Lin Li, Miaomiao Chen, Cheng Wu

**Affiliations:** Department of Gastroenterology, Anhui Provincial Children's Hospital (Children's Hospital of Anhui Medical University), Wangjiang East Road No.39, Hefei 230051, China

## Abstract

Duodenal ulcer seriously affects the quality of life and life safety of children, but the pathogenesis of children with duodenal ulcer is still unclear. As an important second messenger in the body, Ca^2+^ participates in the physiological and pathological processes of various diseases. Therefore, transient receptor potential vanilloid type 4 (TRPV4) as one of the channels that mediate Ca^2+^ has attracted widespread attention in recent years. Here, we found that TRPV4 is highly expressed in children with duodenal ulcer and has good diagnostic value through specimens of children with duodenal ulcer, and animal experiments have proved that TRPV4 is also highly expressed in duodenal ulcer mice. In addition, TRPV4 can enhance intestinal permeability, thereby promoting further infiltration of inflammatory factors. In summary, these results indicate that TRPV4 is involved in the occurrence and development of duodenal ulcer. Therefore, this study provides the diagnostic and therapeutic value of TRPV4 in children with duodenal ulcer.

## 1. Introduction

Nowadays, about half of the world's population is known to be infected with *Helicobacter pylori* (*H. pylori*) and is predominantly acquired in early childhood [1, 2]. And *H. pylori* can cause peptic/gastric ulcers in about 10% individuals [3]. *H. pylori* is one of the main causes of duodenal ulcer and chronic gastritis in children [4]. Studies have shown that *H. pylori* infection in children with duodenal ulcer was high (range, 33 to 100%; median, 92%) compared with children with gastric ulcer (range, 11 to 75%; median, 25%) [5]. Therefore, it is important to find an effective way to diagnose and treat duodenal ulcers.

Calcium (Ca^2+^) is a second messenger regulating a wide variety of function in cells including adhesion, activation, proliferation, and migration [6]. Transient receptor potential vanilloid type 4 (TRPV4), regulating calcium homeostasis, is a member of the transient receptor potential (TRP) superfamily of nonselective cation channels which has been found in many different epithelial cells [7, 8]. TRPV4 was also expressed in intestinal epithelial cells, which can cause the occurrence of inflammatory disease of gastrointestinal tract [9]. There are many research reported that TRPV4 can regulate the function of epithelial barrier though calcium influx. For example, Huang et al. reported that TRPV4 can affect the intestinal epithelial barrier [7]. But whether TRPV4 is involved in mediating duodenal ulcers in children remains unclear.

In the present study, we tested the expression of TRPV4 in duodenal ulcers in children. We also investigated whether TRPV4 can regulate the epithelial barrier in mouse model of duodenal ulcer.

## 2. Methods and Materials

### 2.1. Patients and Animals

The children who recurrent abdominal pain and other gastrointestinal symptoms (vomiting, nausea, loose stools, and constipation) were scheduled for a gastroduodenoscopy. 11 cases of duodenal bulb ulcers were seen endoscopically (with *H. pylori* infection confirmed by rapid urease test) (6 boys; mean age, 10.4 ± 2.1 years; range, 3 to 14 years), and 11 specimens with the above symptoms and mucosal edema of the duodenal bulb seen microscopically (no inflammation by pathological examination) were used as control group (6 boys; mean age, 12.6 ± 4.3 years; range, 4 to 16 years). This study was approved by the Ethics Committee of the Provincial Children's Hospital of Anhui Medical University. Informed consent to participate was obtained from children parents.

Male C57BL/6 J mice (age, 5 weeks; weight, 20–25 g) were purchased from Shanghai SLAC Laboratory Animal Co., Ltd. (Shanghai, China) (no. 201721225; no. 201808847; no. 201807987). Mice were housed in a well-ventilated holding room with a 12 h light-dark cycle at an ambient temperature of 23 ± 2°C and 70% humidity. They had free access to water and food. All animal studies were approved by the Animal Care Committee of Anhui Medical University (Hefei, China).

### 2.2. Experimental Design

Overall, animal experiments were conducted three times. Each experiment, the mice were divided into two groups of 9 animals each. In the first group, the animals received saline (1 ml/kg PO) at 8 AM and 12 PM, and the second group animals received cysteamine (450 mg/kg PO) (Aladdin, C106201, Shanghai, China) at 8 AM and 12 PM [10]. After 24 h, instill the TRPV4 agonist GSK1016790A (GSK, 0.1 mg/kg) (MCE, HY-19608, Shanghai, China) [11] or the inhibitor HC067047 (HC, 1 mg/kg) (MCE, HY-100208, Shanghai, China) [12] by gavage, three mice per group, and the other three with solvent (0.9% saline, the final volume of gavage is 100 *μ*l per mice for each group). After 24 h, all animals were euthanized under ether anesthesia, and the duodena and blood were removed carefully.

### 2.3. Immunohistochemistry

Duodenal tissue specimen was fixed with 4% paraformaldehyde and embedded in paraffin. The specimens were cut into five *μ*m-thick sections, deparaffinized, and dehydrated. Antigen retrieval was accomplished by heating the sections in citrate buffer in a microwave oven for 15 min. The sections were then incubated with hydrogen peroxide (3%) for 30 min to destroy endogenous peroxidase activity. Subsequently, the sections were incubated with primary antibody to TRPV4 (Alomone labs, America) overnight at 4°C prior to incubation with an anti-rabbit secondary antibody (Biosharp, Hefei, China). After being treated first with horseradish peroxidase and then with 3,3′-diaminobenzidine, the sections were counterstained with hematoxylin, dehydrated, cleared, and mounted. For the negative control group, the primary antibody was omitted. Images of stained sections were captured using a light microscope and analyzed with Image Pro Plus 5.1 (Media Cybernetics, Rockville, MD, USA) software [13].

### 2.4. HE Staining

The sections were dewaxed and hydrated. The sections were then stained with hematoxylin solution for 5 min at room temperature. After differentiation with 1% acid alcohol for 1 min, the sections were incubated with 1% eosin for 15 s.

### 2.5. ELISA Analysis

The levels of inflammatory and oxidative stress related indicators and tumor necrosis factor-*α* (TNF-*α*) in samples were detected by commercial ELISA kit (Elabscience, Wuhan, China) according to the product manual.

### 2.6. [Ca^2+^]_i_ Measurement

Carefully separate the mouse duodenal epithelial tissue and fix it on the slide. Then slide with tissues was incubated for 40 min in the dark at 37°C with 6 *μ*M Fluo-8/AM (ABCAM, ab142773, U.K) and 0.02% pluronic F-127 (Sigma-Aldrich, P2443, St. Louis, MO) in the culture media. Next, the tissues were washed with a normal physiological saline solution (NPSS, 140 mM NaCl, 5 mM KCl, 2 mM CaCl_2_, 1 mM MgCl_2_, 10 mM Glucose, and 5 mM HEPES, pH 7.4) and mounted onto a microscope chamber with NPSS and incubated at 37°C. The fluorescent [Ca^2+^]_i_ signals were recorded by a fluorescence microscope (Nikon, Tokyo, Japan). To observe how the TRPV4 agonist GSK1016790A influenced Ca^2+^ release, 50 nM GSK1016790A was added to the cells in NPSS. Changes in [Ca^2+^]_i_ are presented as the ratio of fluorescence relative to the intensity at the beginning (F1/F0).

### 2.7. Intestinal Permeability

The intestinal epithelial integrity is checked by measuring the permeability of fluorescein. Mice were fasted for 2 hours but allowed free access to water and then given fluorescein isothiocyanate-dextran (FD20, 20,000 Daltons, Sigma-Aldrich, St. Louis, MO) (60 mg/ml) with gavage needle 4 hours before euthanized. Blood samples (~0.2 ml each) were collected after 4 h by abdominal aorta. Centrifuge at 3000 g 4°C for 10 min and collected serum. Measurements were made using a FlexStation (Molecular Devices, America). Samples were diluted with 180 *μ*l phosphate-buffered saline (PBS), and fluorescence was measured.

## 3. Results

### 3.1. Significantly Increased Expression of TRPV4 in Children with Duodenal Ulcer and Had Good Diagnostic Value in Children with Duodenal Ulcer

**Figure 1:**
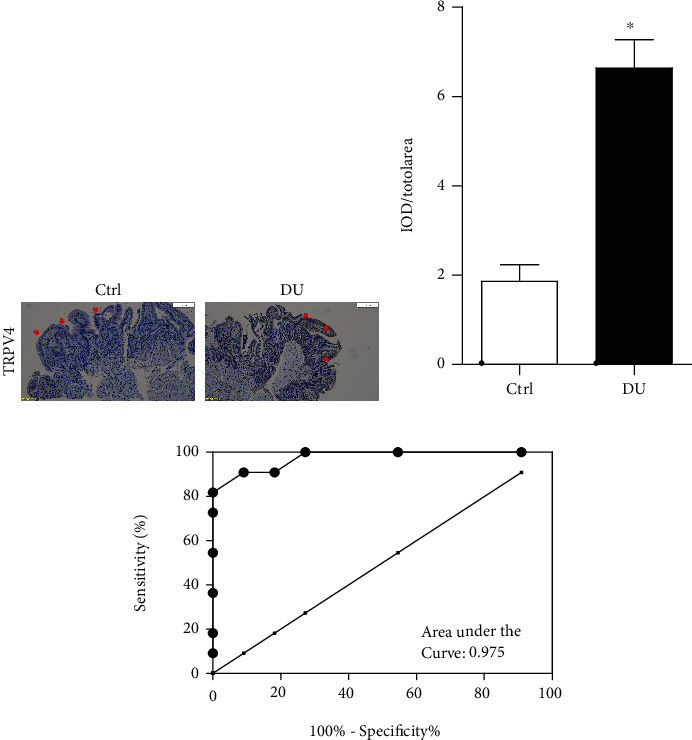
TRPV4 is highly expressed in children with duodenal ulcer and has good diagnostic value. (a) and (b) Immunohistochemistry representative images (a) and summary (b) data showing the expression of TRPV4 in children with duodenal ulcer; Ctrl: nonduodenal ulcer; DU: duodenal ulcer. (c) ROC curve analyses of TRPV4. *n* = 11, ^∗^*P* < 0.05 compared with Ctrl.

In order to prove whether the expression of TRPV4 is different in children with duodenal ulcer, we collected tissue samples from children with duodenal ulcer (DU) and those with nonulcer (Ctrl) and detected the expression of TRPV4 in children with duodenal ulcer through immunohistochemistry. The results suggested that TRPV4 is significantly highly expressed in the tissues of children with duodenal ulcer (Figures 1(a) and 1(b)). In addition, receiver operating characteristic curve (ROC) analysis showed that TRPV4 had a good diagnostic value in children with duodenal ulcer (Figure 1(c)). These results remain us that TRPV4 is highly expressed in children with duodenal ulcer and has diagnostic value and TRPV4 maybe a diagnosis and treatment target for children with duodenal ulcer.

### 3.2. Increased TRPV4 Expression and Enhanced Calcium Influx in Duodenal Ulcer Mice

**Figure 2:**
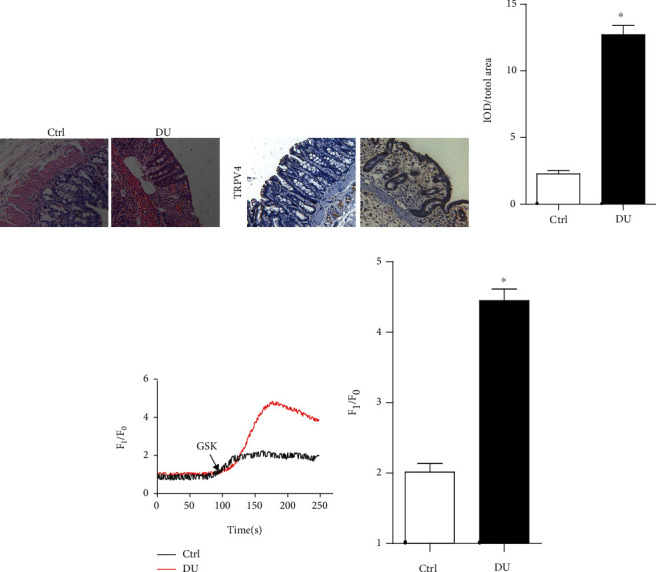
Increased TRPV4 expression and enhanced calcium influx in duodenal ulcer mice. (a) Representative images showing hematoxylin-eosin staining of duodenum tissue. (b) and (c) Representative images (b) and summary (c) data showing the expression of TRPV4 in duodenal ulcer mice. (d) and (e) Representative traces (d) and summarized data (e) showing the changes in the intracellular Ca^2+^ concentration of duodenal tissue between DU and Ctrl group. Ctrl: nonduodenal ulcer; DU: duodenal ulcer. *n* = 3, ^∗^*P* < 0.05 compared with Ctrl.

To further explore the mechanism of DU, we successfully prepared duodenal ulcer mice (Figure 2(a)). We also tested the expression of TRPV4 in the duodenum of mice with duodenal ulcer, and the results showed that TRPV4 was highly expressed in the duodenum of mice with ulcers compared with control group (Figures 2(b) and 2(c)). As we all know, TRPV4 can mediate calcium influx. Therefore, we used GSK1016790A, TRPV4 agonist, to treated duodenal tissue. The calcium image results showed that DU group had a stronger influx of calcium ions after GSK1016790A treated compared with control group (Figures 2(d) and 2(e)). These results remain us that TRPV4 also had a high expression and enhanced function in duodenal ulcer mice.

### 3.3. TRPV4 Channel Can Affect Intestinal Epithelial Cell Permeability

**Figure 3:**
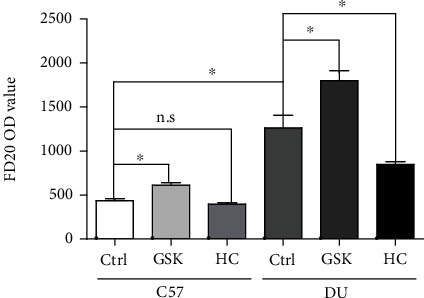
FD20 content in serum. In Ctrl and DU groups, after treatment with TRPV4 agonist GSK1016790 A or inhibitor HC067047, the content of FD20 in serum was detected. Ctrl: nonduodenal ulcer; DU: duodenal ulcer. *n* = 3, ^∗^*P* < 0.05.

Intestinal epithelial barrier is an important factor in inflammatory bowel disease [7]. To explore whether the permeability of the intestinal epithelium has changed in duodenal ulcer disease and the effect of TRPV4 in changing intestinal permeability. We use duodenal ulcer mice to study this problem. As Figure 3 suggested that intestinal epithelial permeability increases significantly in duodenal ulcer compared with control group. In addition, no matter in the control group or the DU group, the TRPV4 agonist GSK1016790A can significantly increase intestinal epithelial permeability. On the contrary, in the DU group, TRPV4 inhibitors HC067047 can significantly reduce the permeability of the intestinal epithelium. These results show that after duodenal ulcer, the increase in intestinal epithelial permeability may be due to the increase in TRPV4.

### 3.4. TRPV4 Channel Can Affect the Content of Inflammatory Factors in Serum, but Does Not Affect the Expression of Inflammatory Factors in Tissues

**Figure 4:**
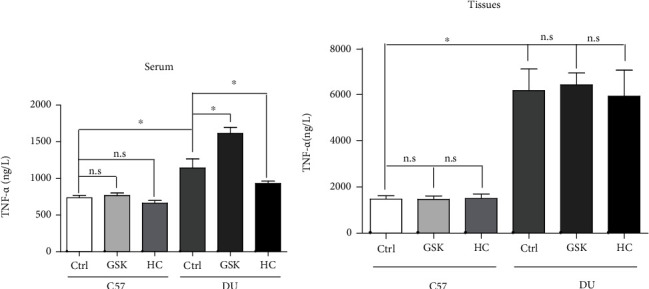
The content of inflammatory factor TNF-*α* in tissues and serum. (a) In the Ctrl and DU groups, serum TNF-*α* content changes after treatment with TRPV4 agonists GSK1016790 A or inhibitor HC067047. (b) In the Ctrl and DU groups, duodenal tissue TNF-*α* content changes after treatment with TRPV4 agonists GSK1016790 A or inhibitor HC067047. Ctrl: nonduodenal ulcer; DU: duodenal ulcer. *n* = 3, ^∗^*P* < 0.05.

Intestinal epithelial barrier is important to prevent harmful substances from further damaging the underlying tissues, including bacteria, toxins, and inflammatory factors [14, 15]. Therefore, we tested the inflammatory factors of tumor necrosis factor *α* (TNF-*α*) in tissue and serum of DU mice and control group. As the results showed that TNF-*α* had a high expression in DU group compared with control group. Moreover, TRPV4 agonists GSK1016790A can significantly increase the expression level of TNF-*α* in serum. Similarly, TRPV4 inhibitors HC067047 can significantly inhibit the expression level of TNF-*α* in serum (Figure 4(a)). But, in the duodenal tissue of duodenal ulcer mice, although the expression of TNF-*α* increased, TRPV4 did not affect the expression of TNF-*α* in the tissue (Figure 4(b)). These results suggested us that TRPV4 can regulate the permeability of intestinal epithelium to regulate the further invasion of inflammatory factors, thereby affecting the progression of duodenal ulcer.

## 4. Discussion

Duodenal ulcer is a refractory disease, which also occurs in children, which seriously affects people's life safety and quality of life [16, 17]. More and more evidence suggests that intestinal permeability may be significant in intestinal inflammatory disease [18–20]. As an important second messenger in the body, calcium ions participate in a series of physiological and pathological processes [21]. TRPV4 is an important regulating calcium channel, especially in epithelial cells [7, 22]. Here, we found that the expression of TRPV4 was significantly increased in children with duodenal ulcers, and it may affect the infiltration of inflammatory factors by affecting the permeability of intestinal epithelium, thereby affecting the development of duodenal ulcer. The major findings of this study are as follows: (1) the expression of TRPV4 was significantly increased in children with duodenal ulcer and had good diagnostic value in children with duodenal ulcer. (2) TRPV4 expression increased and calcium influx also increased in duodenal ulcer mice. (3) TRPV4 can promote intestinal epithelial permeability in mice with duodenal ulcer. (4) Intestinal permeability mediated by TRPV4 can affect the entry of cytokines into the blood and exacerbate the progression of ulcers. In a word, we used clinical samples of children with duodenal ulcer revealed high expression of TRPV4, and the mouse model of duodenal ulcer proved that TRPV4 promotes the occurrence and development of duodenal ulcer by affecting intestinal epithelial permeability.

*Helicobacter pylori* infection has been recognized as a major etiology for the development of duodenal ulcer in children [23]. But not all duodenal ulcers are caused by *H. pylori* infection [24]. Duodenal ulcer is extremely harmful and can easily cause complications such as hemorrhage. Therefore, it is important to explore its specific pathogenesis and find specific therapeutic targets. Nowadays, the treatment of the duodenum is mainly antibacterial treatment [25]. But due to the massive use of antibiotics, drug-resistant *H. pylori* gradually emerged. Finding new treatment methods and treatment targets is essential. In our research, we found that TRPV4 is significantly highly expressed in children with duodenal ulcer and has a good diagnostic value. This discovery can provide new clues and targets for the diagnosis and treatment of children with duodenal ulcer.

TRPV4 is a nonselective cation channel and is expressed in intestinal epithelial cells [10]. Activation of TRPV4 caused an increase in the intracellular Ca^2+^ concentration through influx of extracellular Ca^2+^, triggering an event which is accompanied by a downregulation of the tight junctional proteins claudin -1, -3, -4, -5, -7, and -8 and by dramatic changes in tight junction morphology, including frequent large breaks in the tight junction strands [26]. In addition, calcium/calmodulin-dependent protein kinase can enhance the phosphorylation of adhesin proteins such as VE-cadherin and resulting in disruption of epithelial cell junctions [27]. The activation of TRPV4 in the gastrointestinal tract causes experimental colitis in mice, which also causes the downregulation of claudin-7 and changes in tight junction morphology [22]. In our study, we also found that duodenal ulcer mice have increased intestinal permeability. But it can enhance intestinal permeability, after inhibiting TRPV4. As we all know, intestinal epithelial barrier is very important to prevent the infiltration of some harmful substances, such as inflammatory factors [28]. In the present study, we found that neither the TRPV4 agonist GSK1016790A nor the TRPV4 antagonist HC067047 altered inflammatory factors in duodenal ulcer mice tissue. However, after activating TRPV4, the expression of inflammatory factors in serum is more, and inhibiting TRPV4 can significantly reduce the expression of inflammatory factors in serum. These results indicate that TRPV4 can increase intestinal permeability, leading to further infiltration of inflammatory factors.

## 5. Conclusion

In summary, the present study demonstrated that TRPV4 is highly expressed in patients with duodenal ulcer. Animal models prove that TRPV4 can affect intestinal permeability, thereby promoting further infiltration of inflammatory factors and further lead to the development of duodenal ulcer.

## Data Availability

The data used to support the findings of this study are available from the corresponding author upon request.
